# Structural and Functional Characteristics of miRNAs in Five Strategic Millet Species and Their Utility in Drought Tolerance

**DOI:** 10.3389/fgene.2020.608421

**Published:** 2020-12-08

**Authors:** Animikha Chakraborty, Aswini Viswanath, Renuka Malipatil, Abhishek Rathore, Nepolean Thirunavukkarasu

**Affiliations:** ^1^Genomics and Molecular Breeding Lab, Indian Council of Agricultural Research-Indian Institute of Millets Research, Hyderabad, India; ^2^Statistics, Bioinformatics and Data Management, International Crops Research Institute for the Semi-Arid Tropics, Hyderabad, India

**Keywords:** microRNAs, millets, drought stress, conserved genes, functional genes

## Abstract

Millets are the strategic food crops in arid and drought-prone ecologies. Millets, by virtue of nature, are very well-adapted to drought conditions and able to produce sustainable yield. Millets have important nutrients that can help prevent micro-nutrient malnutrition. As a result of the adverse effect of climate change and widespread malnutrition, millets have attained a strategic position to sustain food and nutritional security. Although millets can adapt well to the drought ecologies where other cereals fail completely, the yield level is very low under stress. There is a tremendous opportunity to increase the genetic potential of millet crops in dry lands when the genetics of the drought-tolerance mechanism is fully explained. MicroRNAs (miRNAs) are the class of small RNAs that control trait expression. They are part of the gene regulation but little studied in millets. In the present study, novel miRNAs and gene targets were identified from the genomic resources of pearl millet, sorghum, foxtail millet, finger millet, and proso millet through *in silico* approaches. A total of 1,002 miRNAs from 280 families regulating 23,158 targets were identified using different filtration criteria in five millet species. The unique as well as conserved structural features and functional characteristics of miRNA across millets were explained. About 84 miRNAs were conserved across millets in different species combinations, which explained the evolutionary relationship of the millets. Further, 215 miRNAs controlling 155 unique major drought-responsive genes, transcription factors, and protein families revealed the genetics of drought tolerance that are accumulated in the millet genomes. The miRNAs regulating the drought stress through specific targets or multiple targets showed through a network analysis. The identified genes regulated by miRNA genes could be useful in developing functional markers and used for yield improvement under drought in millets as well as in other crops.

## Introduction

MicroRNAs (miRNAs) are small, single-stranded, non-coding, endogenous RNA of size varying from 21 to 24 nucleotides mainly involved in post-transcriptional gene regulation (Zhang et al., 2014; Aravind et al., 2017). They are highly conserved in matured form, and the conserved nature has made it a molecule of interest in several plant growth, development, and stress regulatory studies without any species boundaries. miRNAs regulate gene expression by targeting specific genes that are involved in biological processes, such as development and metabolic process, as well as target-specific transcription factors (TFs). Interaction between the miRNA–mRNA target is more important as it induces variation in the gene being expressed. Regulation of gene expression occurs in several ways, such as miRNA-directed mRNA cleavage, translational repression, chromatin remodeling, or epigenetic modification (Kumar et al., 2018). Single miRNA itself can target several genes involved in the same cellular signaling pathway (Alptekin et al., 2017).

Studies have shown that miRNAs and their targets are highly conserved across all major lineages of plant species, including dicots and monocots (Jones-Rhoades and Bartel, 2004). There are at least four theories about the origin of miRNAs: (1) inverted duplication events in the gene sequences; (2) duplication events from the protein-coding genes; (3) derived from the transposable elements, such as miniature inverted-repeat transposable elements (MITEs); and (4) accumulation of mutations in the inverted repeats and selection (Cuperus et al., 2011; Nozawa et al., 2012). Evolutionarily conserved miRNAs are mostly encoded by gene families. This, coupled with miRNA–mRNA target interaction, results in overlapping functions of miRNAs belonging to the same families (Jones-Rhoades et al., 2006).

miRNA families can be divided into two categories, based on the nature of their conservation and function. The first type is ancient in terms of evolution, highly conserved in the system with a high degree of expression. Conserved miRNAs are ubiquitous with low sequence variation and play an important role in basic biological functions through regulation of transcription factors and genes. The second type is relatively young in terms of evolution and expresses only when induced under specific conditions. Since they recently evolved to perform a specific function, the sequence variation is high in such types (Qin et al., 2014). Even though miRNA families are conserved among plants, they are more specific to the species of plant, physiological stages, type of organ/tissues, and stress conditions (Sun, 2012; Banerjee et al., 2016; Sunkar et al., 2017). This conserved nature over long evolutionary distances suggests the role of the evolutionarily conserved mechanism of miRNA in gene regulation (Molnar et al., 2007).

Abiotic stresses negatively impact plant growth, development, and productivity by altering the gene expression patterns. In order to cope with stress conditions, plants have developed several mechanisms over time, including the intricate interactions between stress-responsive elements and various molecular and biochemical factors affecting growth and development (Razmjoo et al., 2008). The plant molecular responses to abiotic stresses involve interactions and crosstalk with many molecular pathways, including miRNA-mediated regulatory pathways (Bej and Basak, 2014). miRNA-mediated regulation involves a change in self-concentration and modifying the mRNA expression. These regulations, in turn, change the protein expression when exposed to stress (Ding et al., 2009; Wang et al., 2014).

Under drought stress, plants have evolved a series of protective mechanisms to withstand adverse conditions (Jaworski et al., 2010). Plants produce an array of gene regulation responses, which include triggering the expression of several stress-related genes, accumulation of osmotically active metabolites, and biosynthesis of specific proteins (Nepolean et al., 2014; Mittal et al., 2017a,b). Many studies have shown that the miRNAs were important modulators of drought tolerance in plants, where they modify the translation of target mRNAs that contain sequences that are complementary to the mature miRNAs. A study conducted in wild emmer wheat produced differential expression patterns of 13 miRNAs in response to drought stress (Kantar et al., 2011; Aravind et al., 2017).

Millets are a group of small grain crops of the family Poaceae, widely grown in the arid and semi-arid tropical regions of Asia and Africa. They are highly favored for food sustainability, owing to climate-resilient features, such as diverse adaptation to arid, semi-arid, and humid conditions. They tend to be less prone to biotic and abiotic stresses, and they can be grown in marginal lands (Kole et al., 2015). Compared to other cereals, millets show exceptional tolerance toward diverse abiotic stresses including drought, salinity, and heat stresses (Bandyopadhyay et al., 2017). In earlier studies on pearl millet, miRNAs were identified using non-pearl millet genomes (Jaiswal et al., 2018; Kumar et al., 2018). miRNAs in sorghum for drought (Katiyar et al., 2015; Hamza et al., 2016) and foxtail for drought (Wang et al., 2016) and dehydration stress (Yadav et al., 2019) using NGS approaches gave rise to a group of differentially regulating miRNAs. In our experiment, we selected five important millet species, namely pearl millet, sorghum, foxtail millet, finger millet, and proso millet, to mine the miRNAs using the latest genomic resources and *in silico* approaches and to track their stress-responsive features at the cellular, molecular, and physiological levels. We structurally characterized the miRNAs and identified conserved domains, such as sequence signatures in mature miRNAs across millet species. We identified target mRNAs regulated by the miRNAs and annotated the functional genes and transcription factors. We explained comprehensively how specific miRNAs regulating various genes in response to drought stress can be used to increase the productivity of millets and other crops.

## Materials and Methods

### miRNA Reference and Genomic Data

Known microRNAs and their precursor sequences from different plants were obtained from miRNA databases PNRD (Yi et al., 2015) and miRBase (http://www.miRbase.org/). Of the 16,436 miRNAs we obtained, a total of 5,906 plant miRNAs served as reference after removal of the redundant sequences. Most of these miRNAs were identified or verified by experiments, and others were computationally predicted as their close homologs.

Genomic sequences in the form of expressed sequence tags (ESTs), genome survey sequences (GSS), and whole-genome sequences (WGS) were retrieved from the NCBI (http://www.ncbi.nlm.nih.gov/) for five millet species: pearl millet [*Pennisetum glaucum* (L.) R. Br.], 5265 ESTs, 4105 GSS, and WGS; sorghum [*Sorghum bicolor* (L.) Moench], 80461 GSS and WGS; finger millet [*Eleusine coracana* (L.) Gaertn.], 2021 ESTs; foxtail millet [*Setaria italica* (L.) Beauv.], 66052 ESTs and 96975 GSS; and proso millet (*Panicum miliaceum* L.), 216 ESTs. The reference genome sequence assembly accessions used in our study for pearl millet, sorghum, finger millet, foxtail millet, and proso millet are GCA_002174835.2, GCF_000003195.3, GCA_002180455.1, GCA_000263155.2, and GCA_002895445.2, respectively (https://www.ncbi.nlm.nih.gov/assembly).

### Pre-processing

Distinct pre-processing steps were taken to draw miRNA candidate sequences from the respective millet genomes. The BLAST version 2.6.0+ alignment tool was used for BLASTn to find the homologs. All known hairpin loop sequences were used as reference in the BLAST search against the genome with accurate parameters as follows: word-size 11 and E value cut-off 10^−3^ and 100 percent identity value, with a maximum three mismatches allowed and default settings for the remaining parameters. Flanking regions from both sides of the matched sequences were cut to a length of 70 nt and scanned by a sliding window of 100 nt (Wang et al., 2005). Duplicated sequences were discarded, and the remaining query sequences were searched using protein and nucleotide databases filtered to remove the presence of rRNA, tRNA, and mRNA, leaving the candidate sequences to be treated as miRNA precursors.

### Structure Prediction

The secondary structure of the candidate precursor sequences was predicted by RNA fold (Hofacker et al., 1994) using the Vienna R package (Lorenz et al., 2011). The folding structures prediction uses the minimum free energy (MFE) algorithm and base pairing probability matrix. Sequences satisfying the criteria are as follows: (1) precursors having no more than three mismatches with previously known plant miRNAs; (2) the secondary structure should be folding into a perfect or near-perfect stem loop hairpin; (3) the mature sequence should be located in one of the arms of the stem loop; (4) presence of loop in the miRNA sequence is not allowed; (5) only the sequences with MFE lower than −20 kcal/mol are kept, and (6) A+U nucleotide content is 25–70% (Patanun et al., 2013).

The miRNAs position on the stem loop structure were predicted by the MirDup (Leclercq et al., 2013), using the training plant model for the candidate mature miRNAs. The classifier uses 10-fold cross validation. The ranking method was performed using the information gain evaluator in WEKA. The MirDup uses the random forest classifier trained with an unlimited maximum depth of the trees.

### Potential Target Identification

Putative gene targets were identified by complementarity between miRNA and mRNA sequence. The targets against all miRNAs of five millet species were identified using the plant miRNA analysis psRNA Target Tool (http://plantgrn.noble.org/psRNATarget/analysis) (Dai and Zhao, 2011). The genome and EST sequences of the crops were taken as input, considering the seed region 2–13 nt, maximum UPE 25, and expectation value 5 at the most. miRNA and mRNA complementary sites were scored 0.5 or 1, according to the G:U match or non-match. Additionally, no more than two consecutive mismatches and no more than four mismatches between mature miRNA and potential target (Chai et al., 2015) were allowed. Due to lack of annotation of the millets, protein coding genes were identified from the putative target mRNAs using BLASTx from either the same crop or from other plants, such as *Arabidopsis* or rice.

### Gene Ontology

**Figure 1:**
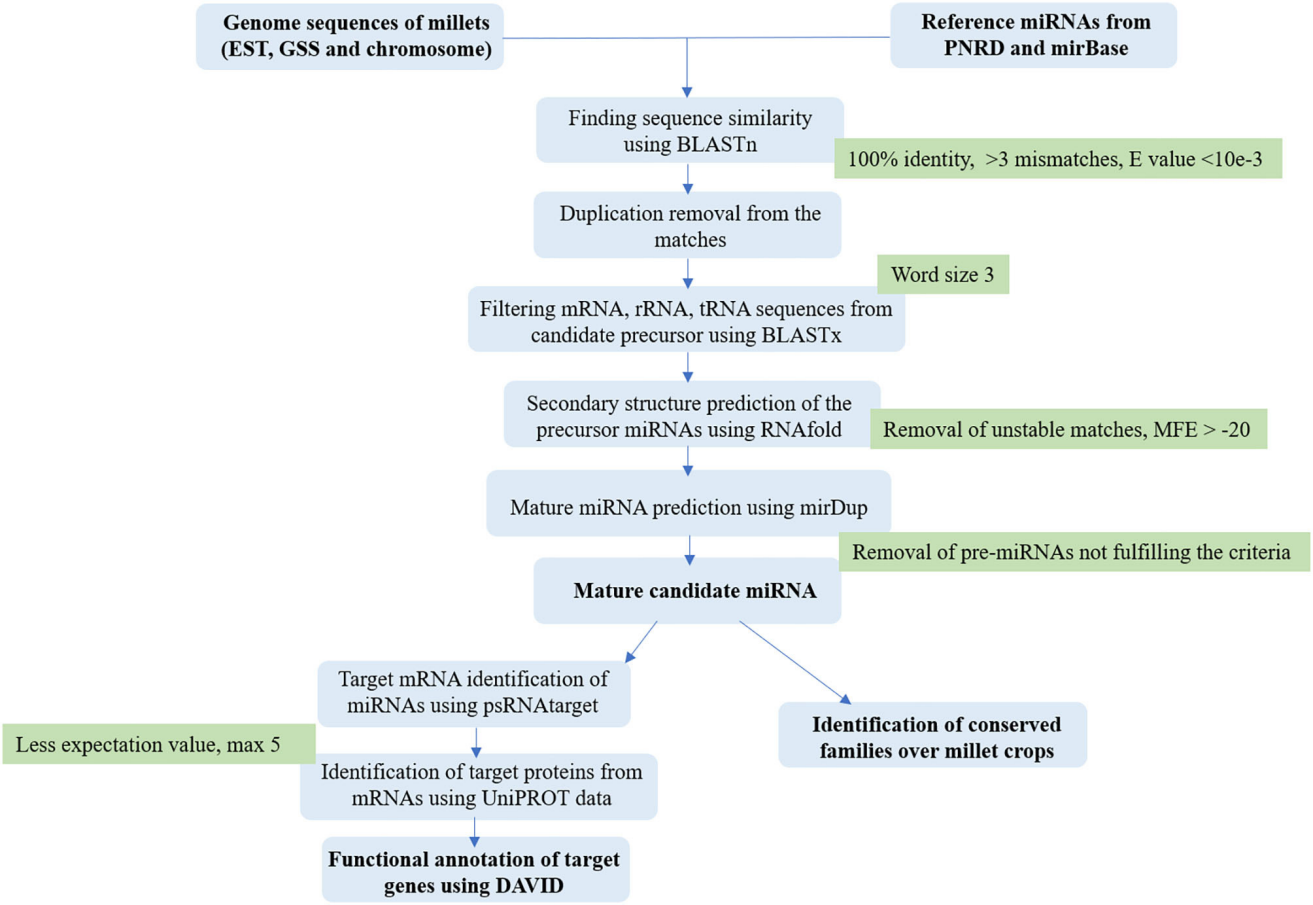
The workflow describes the comprehensive *in silico* pipeline for the identification of miRNAs from millet genomic resources.

The gene ontology (GO) base is continually evolving as biological knowledge increases and the curation of biological process develops. Target genes were subjected to functional annotation to reveal the miRNA-mediated gene regulatory network on biological processes, cellular components, and molecular functions. GO annotation was performed using DAVID (https://david.ncifcrf.gov/) for individual crops to understand the diverse function. The DAVID functional annotation clustering tool provided a module-centric approach for functional analysis of large gene lists. The grouping of data is based on functional categories and co-expression profiles, such as genes in the same pathway. A gene-term matrix gives different categorical clusters, such as GO biological process, GO molecular function, and GO cellular component and pathways (Huang et al., 2007). The calculation of over-representation of GO terms was done by applying the Fisher's exact test for count data and *p*-value (Benjamini and Hochberg, 1995). Figure 1 shows the comprehensive workflow used for miRNA.

### Gene Network

A network of drought targets of the miRNAs was created using Cytoscape (https://cytoscape.org/) (Shannon et al., 2003) to point out the hub genes of drought responsiveness.

## Results

### Identification of Pre-miRNAs

The species-specific miRNAs were found by precise excision of the stem-loop precursor and single strands with a length of ~22 nt (Zhang et al., 2009). After BLASTn search against plant miRNAs from miRbase, the hits without protein coding sequences, tRNAs, and rRNAs were kept for secondary structure analysis. We identified 10,376, 9,064, 8,748, 8,173, and 3,055 homologous sequences in sorghum, foxtail, pearl, finger millet, and proso millet, respectively (Table 1). The homologous sequences were filtered by removing other RNA matches (tRNA, mRNA, rRNA), which resulted in the removal of 8,173, 3,493, 771, 478, and 27 candidate sequences from finger millet, sorghum, foxtail millet, pearl millet, and proso millet, respectively (Table 1).

**Table 1:** Pre- and mature miRNAs identified through *in silico* tools in five millet species.

**Crop**	**Blast matches**	**Filtered-off tRNA, rRNA, and mRNA homologs**	**Entries without duplication**	**Stable match**	**Rejected miRNA candidates**	**Final mature miRNAs**
Finger millet	8,173	8,173	0	75	58	17
Foxtail millet	9,064	771	8,293	1,615	1,242	373
Pearl millet	8,748	478	8,270	808	626	178
Proso millet	3,055	27	3,028	464	21	404
Sorghum	10,376	3,493	6,883	815	781	34

The stability of the hairpin must be high with the lowest free energy of all other alternative folds for that sequence, which were predicted by RNA fold (Mathews et al., 2010). The minimal folding free energy (MFE) index is a major feature to distinguish putative precursor miRNAs from other RNAs. It was observed that more than 90% of the miRNAs had an MFE value <-30 (Zhang et al., 2006). Stable fold structures with MFE −20 were sorted out from the remaining structures producing 1,615, 815, 804, 434, and 75 stable precursor miRNAs from foxtail, sorghum, pearl millet, proso millet, and finger millet, respectively (Table 1).

### miRNA Clusters in Genome

**Figure 2:**
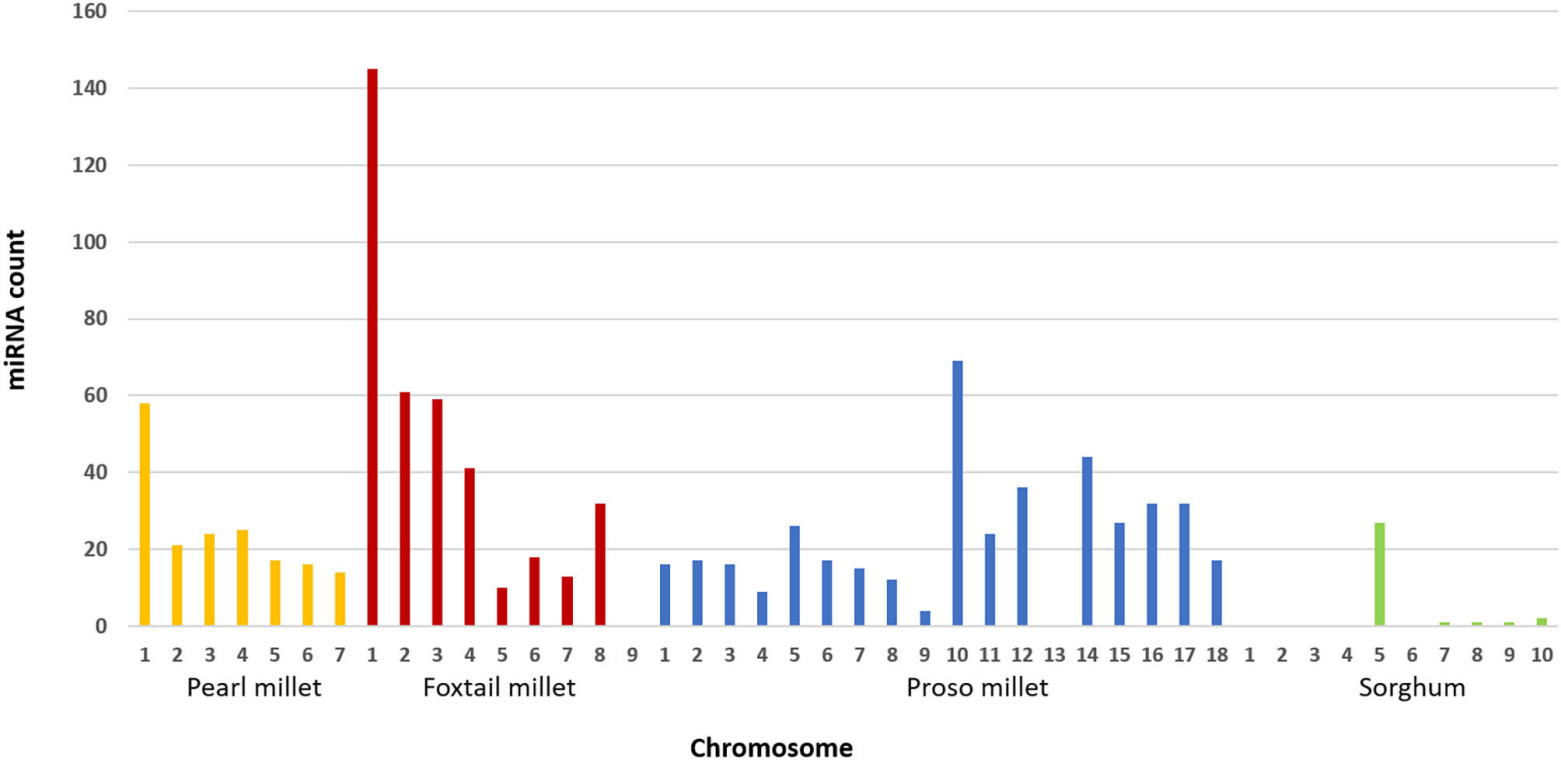
Distribution of miRNAs across chromosomes in pearl millet, foxtail millet, proso millet, and sorghum.

Among the candidate miRNAs, unique mature miRNAs were identified from foxtail millet, pearl millet, proso millet, and sorghum (Figure 2). Of these families, some were frequently present in different chromosomal regions in respective millets. In foxtail millet, a maximum number of miRNAs was observed in chromosome 1 (145 miRNAs), followed by chromosomes 2, 3, and 4 (50 miRNAs). Of the families, miR156 and miR157 were observed 9 times higher in chromosome 1. In pearl millet, miR167 and miR169 were the most frequent families, located in chromosomes 2 and 1, respectively, and chromosome 1 had the maximum miRNA families count among all chromosomes. Chromosome 10 in proso millet was the largest contributor with 68 miRNA families, and the major families were miR396, miR167, and miR166. In sorghum, chromosome 5 had the highest number of families. In finger millet, 14 unique families were identified, but the chromosome-specific miRNA count could not be tagged since genome was distributed in scaffolds.

### Sequence Characteristics of New miRNAs

Transformation from precursor to mature form includes one major feature, the presence of mature 5′ and 3′ stems. Tools, such as Mature Bayes and the MirDup tool were used to bring out the mature form from the stable precursor miRNA molecule (Gkirtzou et al., 2010). A total of 404, 373, 174, 34, and 17 mature miRNAs were obtained from proso millet, foxtail millet, pearl millet, sorghum, and finger millet, respectively (Table 1).

**Figure 3:**
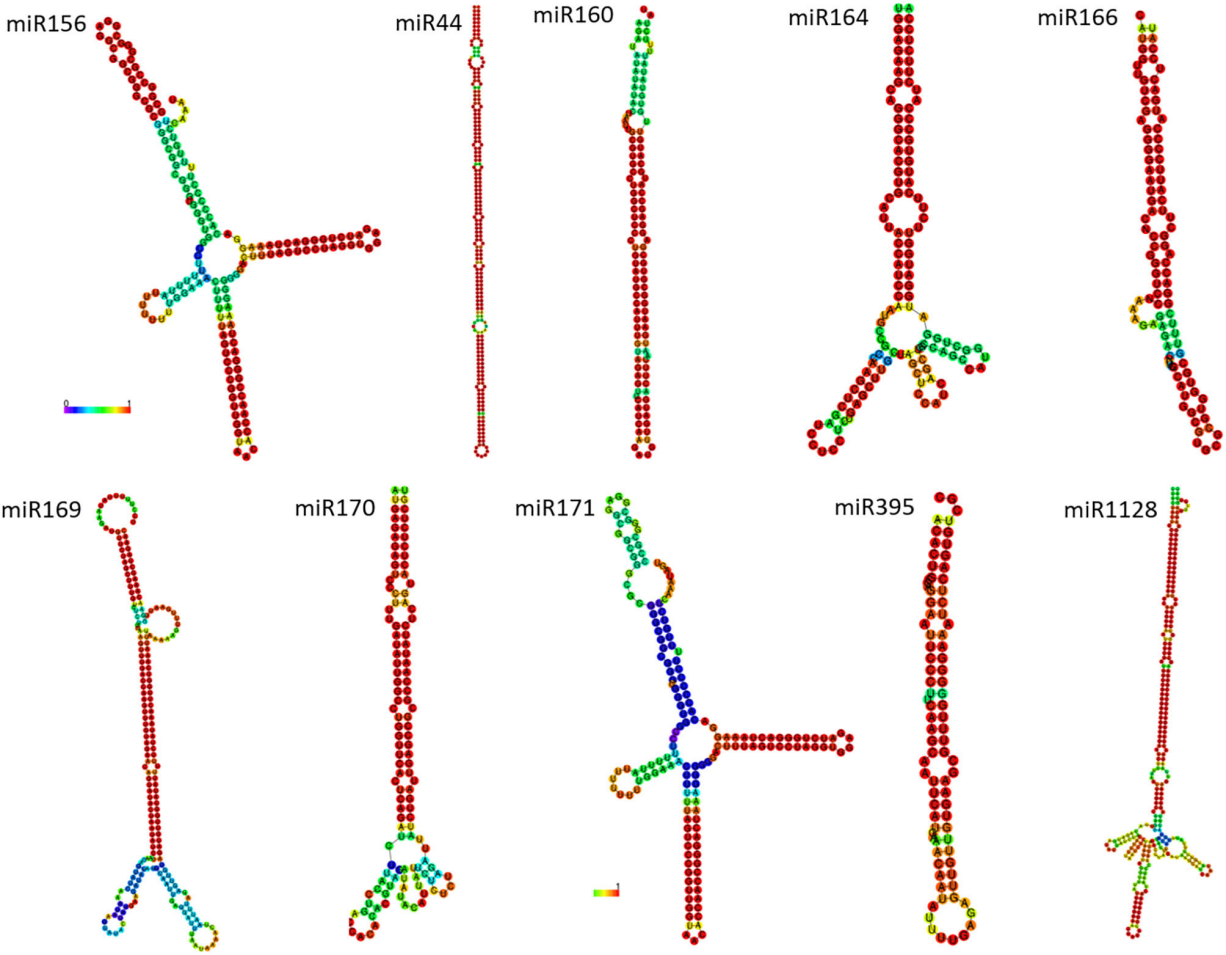
Major miRNA family precursor structure based on MFE values and base–pair probabilities.

The conserved miRNA sequences identified were variable in length across families, and in some families, the members were of uniform size. There were two classes of precursors with different structural properties. The most abundant class included precursors that had only two strongly conserved regions or blocks that consisted of the miRNA and miRNA^*^ (^*^ = complementary sequence of hairpin loop). The foldbacks of these precursors contained a short stem, consisting mainly of the miRNA/miRNA duplex. The consensus structures of miRNA families, such as miR156, miR160, miR170, miR171, miR395, and others are shown in Figure 3. A second and less frequent class, which includes the miRNA families miR159/319 and miR394, displays four conserved sequence blocks (Dezulian et al., 2006).

### Conserved miRNAs Across Millets

**Figure 4:**
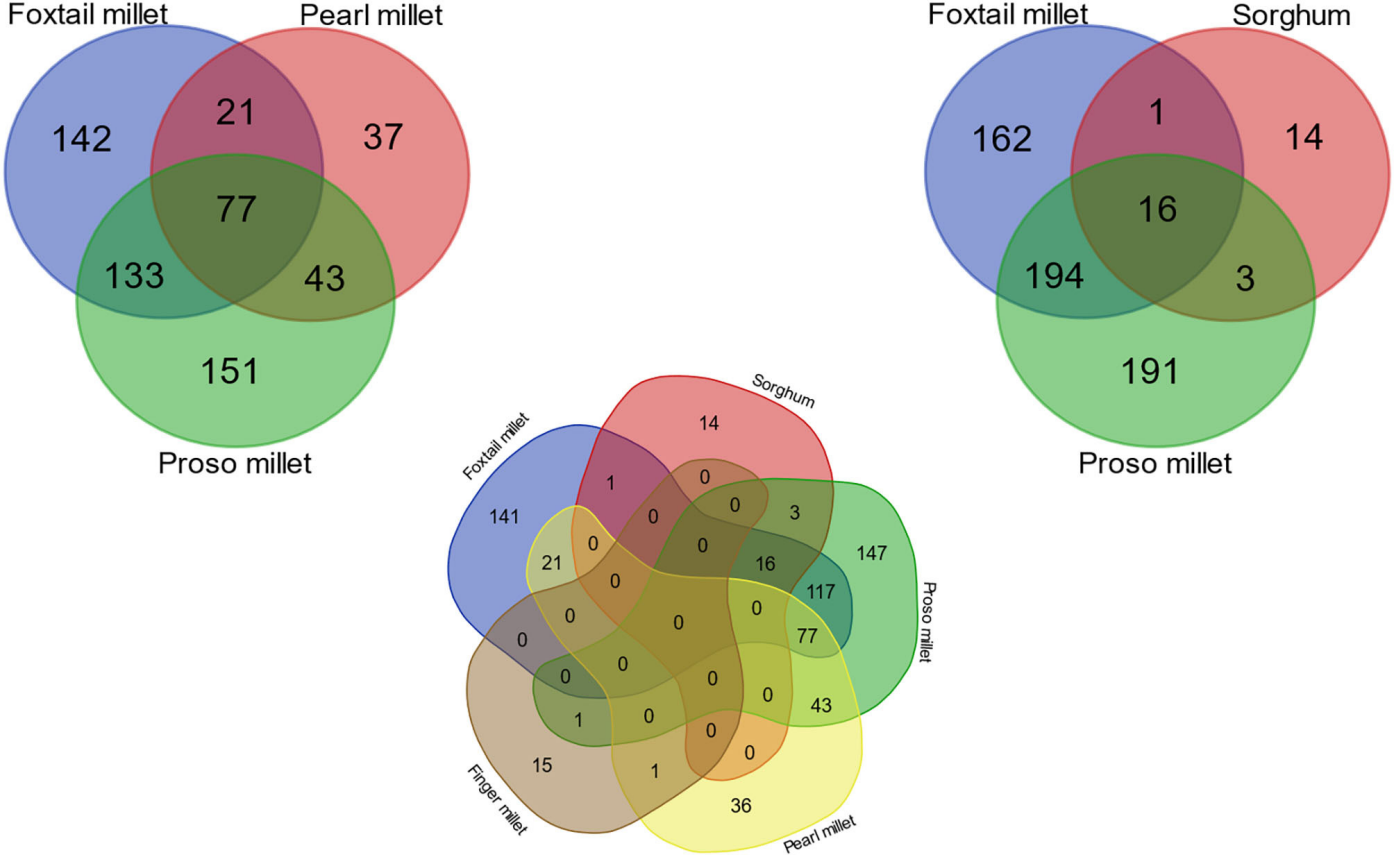
Venn diagram showing common and unique miRNAs among five millet species.

The present study has revealed 77 miRNAs were observed commonly repeating in pearl millet, proso millet, and foxtail millet (Figure 4). A smaller number of identified miRNAs in finger millet was not included in the analysis. Previously known reference miRNAs from sorghum and foxtail millet were also considered, which infers only three were common between old and newly discovered miRNA in sorghum, while for foxtail millet, all the miRNAs were new. The frequently co-occurring families (66) showed a conserved sequence pattern and consensus folding in the secondary structure. In our study, foxtail millet, proso millet, and pearl millet showed 27.9% conserved pattern in identified miRNAs, whereas foxtail and proso millet had more than 50% similarity (Figure 4). About 28–37% of miRNA families were matching among foxtail millet, sorghum, and proso millet. There was no conserved found in all millets together. In finger millet, we found only two miRNA families (miR845 and miR1873) common between pearl millet and proso millet due to incomplete genome and lack of annotation.

### Target Prediction

In all five millets, most of the genes were identified as a result of specific stress conditions, such as drought, cold, salt, and water deficit. A total number of 7,090, 4,063, 1,754, 238, and 121 unique targets were identified in foxtail, sorghum, pearl millet, finger millet, and proso millet, respectively (Table 2). In pearl millet, miR134 acts on translation initiation factor like 5A-1/2, miR164a on heat shock TF, and miR172 on TF SUI1, which are evolutionarily conserved proteins (Koia et al., 2013). Some miRNAs control different physiological functions, such as miR10302, which was observed for regulating MYR1 protein related to flowering time under low light intensity in *Arabidopsis* (Zhao and Beers, 2013) and miR34 functions in seedling salt stress in broccoli (Tian et al., 2014).

**Table 2:** miRNA-mediated genes, transcription factors, proteins, and enzymes identified in the genome of five millet species.

**Feature**	**Foxtail millet**	**Sorghum**	**Proso millet**	**Pearl millet**	**Finger millet**
Genome size (mbp)	423	666	851	1,817	1,195
Total gene target	13,714	5,882	319	2,977	266
Unique gene target	7,090	4,063	121	1,754	238
Transcription factor	902	193	–	24	14
Enzymatic activity	1,445	763	19	87	64
Stress regulatory	62	516	253	621	28
Carrier protein	43	10	21	102	21
Growth/physiological factor	39	–	–	–	92

Out of 121 mRNA targets, 104 played essential roles in stress responses in proso millet, which was mostly targeted by miR44, miR156, miR159, miR160, miR166, miR169, miR171, miR172, and miR177. Enzymes, such as dioxygenase, kinases, dehydrin, and malate translocator operating on heat shock and ripening pathways were controlled by miR81, miR148, and miR164. Sorghum had 4,063 unique mRNA targets, mostly involved in different binding proteins, transcription factors, stress-related HSPs, and dehydration stress-related proteins, along with aminopeptidases and kinases.

In all millets, the miRNAs were mostly targeting the TFs, stress-related genes, metal-deficit factors, enzymes, and pathogenic factors other than normal metabolic and physiological regulators. Although the majority of plant miRNA targets were captured by the cut-off of expectation value <5 and mismatches 0 to 3, several authentic targets were missed due to lack of annotation.

### GO Annotation

**Figure 5:**
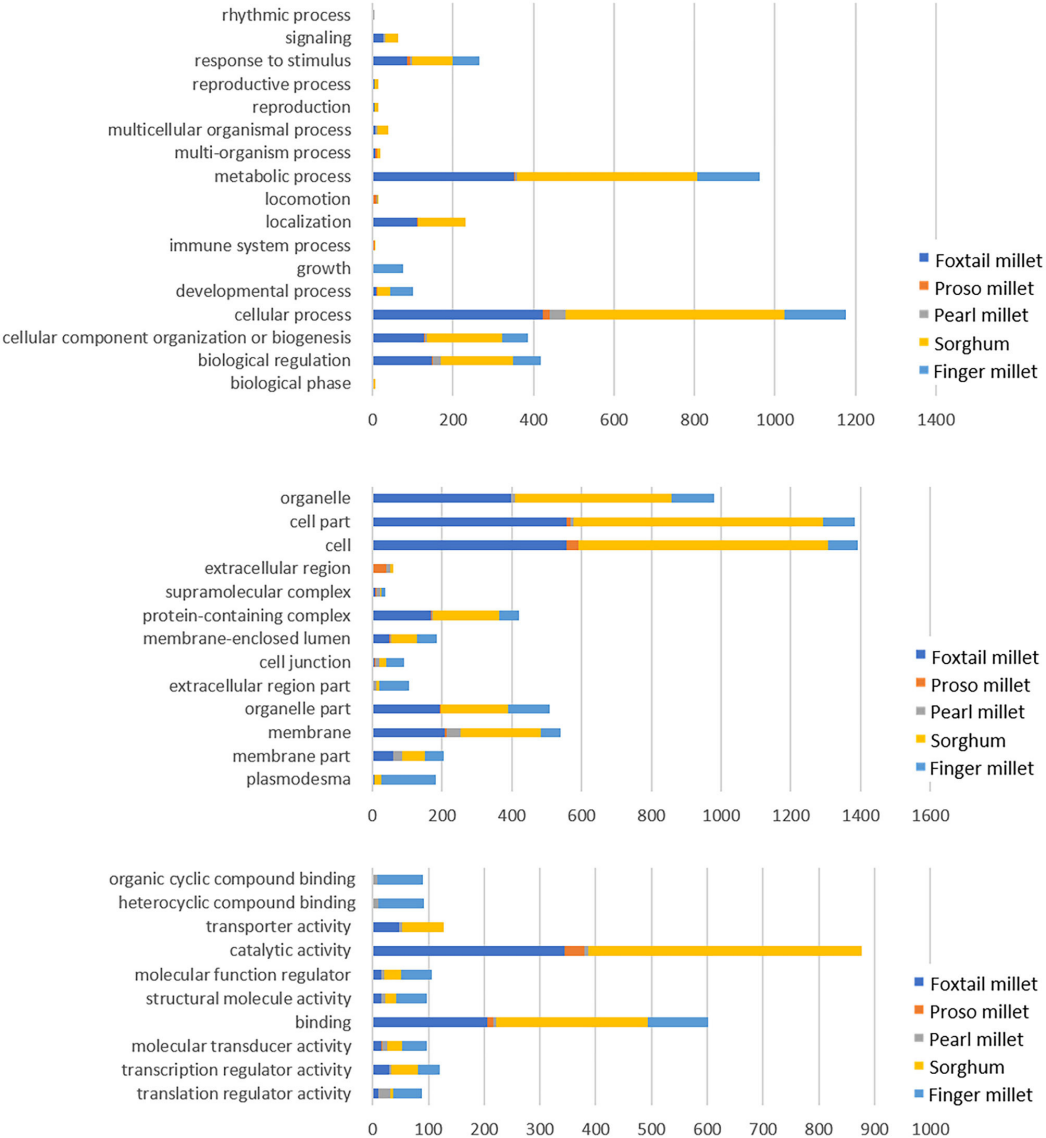
Significantly enriched GO terms for the target genes of the miRNAs across millets.

The target gene sets were subjected to GO analysis, which covers three domains (biological process, cellular component, and molecular function) to interpret the underlying functions of miRNAs (Figure 5). The biological process category showed a maximum number of genes participated in cellular process (545, 424, 153, 150, and 48 in sorghum, pearl millet, foxtail millet, finger millet, and proso millet, respectively), followed by metabolic processes, biological regulation, and cellular component. In cellular component, most of the genes were grouped in the cell, cell part, organelle, and protein-containing complex (2685, 2219, 1281, 102, and 98 genes in sorghum, foxtail, pearl, proso millet, and finger millet, respectively). Molecular function revealed the underlying activity of the genes, such as binding, catalytic activity, transcription regulation, transporter activity, molecular function regulation, and structural molecule activity.

## Discussion

### Conserved miRNAs Across Millets

Identification of conserved miRNAs families on evolutionary basis among plants has provided a powerful approach to understanding gene regulation among related species. Identical miRNA sequences exist in closely and distantly related plant species. Many of the miRNAs discovered were found in a wide range of plant groups, from mosses to angiosperms. Some miRNA families exist broadly within the angiosperms, including eudicots and monocots, dating back to at least the early Cretaceous. Several miRNA families also pre-date the divergence of gymnosperms and angiosperms (305 million years) and the divergence between vascular plants and mosses (490 million years). These results indicated that miRNA sequences are highly conserved across great phylogenetic distances and that similar selection pressures have been active in the regulation of gene expression in plant cells since the earliest stages of their evolution (Zhang et al., 2006).

Evolutionarily conserved miRNAs are mostly encoded by the same gene families, and the members of these families are physically clustered in the entire plant genome (Tanzer et al., 2005). The mature miRNA sequences are highly conserved among the same family members, and this extends to the entire stem-loop precursor duplex. Axtell and Bartel (2005) identified the occurrence of certain miRNA families (miRNA159 and miRNA319) common in ten different plant species and similarly the expression of miRNA165 and miRNA166 in nine plant species. We analyzed each millet with individually discovered potential miRNAs in other millets to find the orthologous candidates. All five millets species belong to same sub-family *Panicoideae*, in which pearl millet, finger millet, foxtail millet, and proso millet belong to the tribe *Paniceae*, while sorghum belongs to another tribe, *Andropogoneae* (Vetriventhan et al., 2020).

Similar to mature miRNA sequences that are conserved across different plant species, the targets are also specific and conserved within the plant families by possessing highly conserved sequences at their complementary sites as we found in millet species. Although there are many nucleotide changes among the targets of different plant species, the sequences of the complementary sites are highly conserved. This is consistent with the study conducted by Floyd and Bowman (2004), in which class III HD-Zip genes targeted by miRNA 166 had specific conserved target regions. It was observed that the sequence similarity of miRNA coupled with specific target results in overlapping functions of miRNAs belonging to the same families (Jones-Rhoades et al., 2006). Families, such as miR156, miR157, miR159, miR165, miR166, miR172, miR390, and so on were highly conserved in all vascular plants studied (You et al., 2017).

miRNAs are highly conserved in the plant kingdom, irrespective of the time of evolutionary divergence. Many families of miRNA are orthologous and homologous in different plant species spanning the breadth of green plant phylogeny (Dezulian et al., 2006). A comparative genomic experiment revealed that finger millet with sorghum produced 69 pairs of syntenic miRNA precursors, which were conserved between them, thereby indicating the evolutionary relationship of miRNA families across different species (Yi et al., 2013). Comparative analysis of miRNAs identified in *A. trichopoda* revealed that several miRNA families orthologous and paralogous with other crops. Among the conserved miRNAs identified in *A. trichopoda*, the miR407 family had three orthologs in *A. thaliana, Zea may*, and *Gossypium hirsutum*. Similarly, there were two orthologs for *A. trichopoda* miR417 with *A. thaliana* and *Oryzae sativa* (Hajieghrari et al., 2015). Relative to sorghum, proso millet and pearl millet are evolutionarily the closest species, with their supposed common ancestor dating back ~27 Mya, followed by ~8.3 Mya between pearl millet and foxtail (Singh et al., 2017). All millets possess considerable morphological differences but are evolutionarily well-related and thus share common structural and functional similarities.

### miRNA and Genomic Targets

Prediction of the mRNA targets of the miRNAs identified from the millets will improve our understanding of the functions and regulation of these miRNAs. The conserved miRNAs, such as miR156, miR166, miR165, miR169, miR393, miR395, miR160, miR170, miR171, and miR172 were identified with 50–100 targets in different millets. We identified 902, 194, 24, and 14 TF targets in foxtail millet, sorghum, pearl millet, and finger millet, respectively, which were related to plant development, phase change in growth, and other molecular functions. A set of 74 miRNA families was identified, targeting different stress regulatory factors, such as drought stress, dehydration stress, oxidative stress, and salt stress. Another important functional group of the predicted targets of miR155, miR156, miR169, miR172, miR2180, and miR2118 families were associated with enzymes, such as kinases, phosphate synthase, acetyltransferase, acid dehydratase, and hydrolase. Most of the miRNA targets were classified into the binding category and appeared to be involved in membrane, steroid, nucleic acid, protein, and ion binding. Around 30 miRNA families were involved in diverse molecular functions, including DNA binding, zinc ion binding, oxidoreductase activity, catalytic activity, protein kinase activity, and transferase activity in all five crops. Our target prediction results confirmed the widely held view that most plant miRNA targets encode TFs, which operate different mechanisms in the millets.

### Drought-Responsive Target Prediction

Our study on identification of miRNA families targeting drought-related factors revealed that 89 targets were found in pearl millet regulated by 29 miRNAs. In proso millet, 64 miRNAs were found targeting mRNAs related to stresses, including drought-related pathways. Around 400 drought-specific mRNA targets were found in sorghum and foxtail millet controlling diverse pathways. In finger millet, 25 mRNAs specific to drought and water-deficient stress were identified to target nine different miRNAs. It was observed that many of the miRNAs families were both functionally and structurally conserved among the species, indicating a broad conservation of the regulatory roles in millets.

### Network of Drought-Responsive miRNA and Their Targets

**Figure 6:**
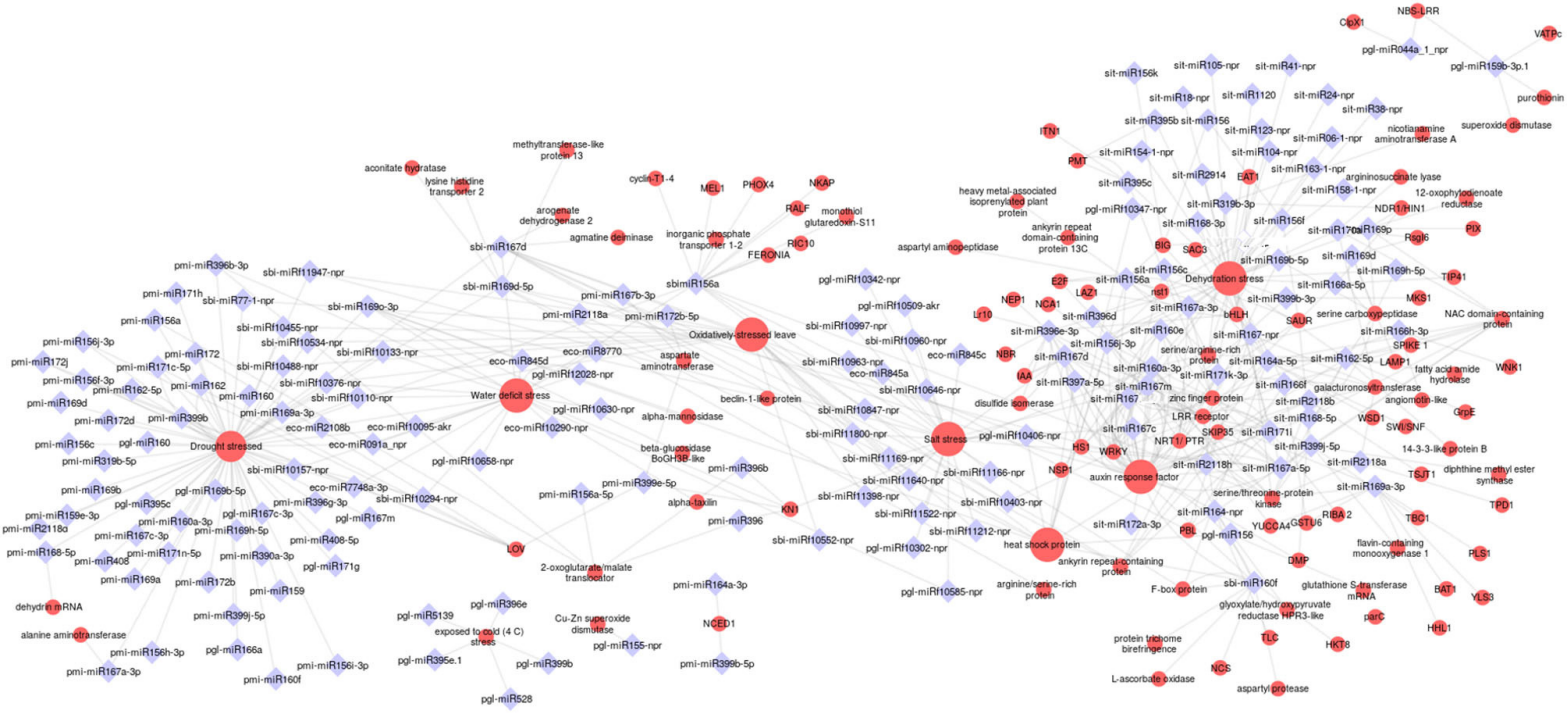
Stress-specific interactions explained through miRNA-gene interconnected networks. The red circle indicates the drought mRNAs, the blue diamond indicates the miRNAs. The size of the red circle indicates the degree of connectivity of the target genes.

Our prediction of target genes revealed that multiple genes can be targeted by one specific miRNA, which suggested that the miRNA research should focus on networks more than on individual connections between miRNA and strongly predicted targets. To investigate the relationship between drought-responsive miRNA and their targets, a network analysis was carried out using the Cytoscape platform. The analysis incorporated all the non-conserved and conserved miRNAs of millet species belonging to 22 families. Targets involved in stress tolerance or plant development, such as genes encoding transcription factors, protein kinases, and phosphatases, and hormone-responsive factors were considered in the network analysis (Figure 6). It was found that the conserved miR156, miR160, and miR167 targeted up to 4 mRNAs in pearl and proso millet, and 20 in sorghum and foxtail millet. miR2108, miR170, and miR171 targeted important genes, such as auxin response factors (ARFs), NTR/PTR, NAC domain, and heat stress factors. Important TFs, such as WRKY and bHLH were regulated by miR399 and miR396 in foxtail and sorghum, respectively. Analysis of the network showed that miR160, miR156 in sorghum, and miR155 in foxtail millet had maximum connectivity. Drought stress–related mRNAs were one of the hubs (high-degree node in network) in the network with 86 connectivity. ARFs and dehydration stress were observed with a connectivity of 46 miRNAs each. Different TFs, such as bHLH, nst1, and WRKY were identified as the semi-hub targets. Six to ten miRNAs were identified as cross-interacting with drought-responsive factors and ARFs and water-deficit stress. Approximately 10 cross-talking miRNAs were identified between dehydration stress and TFs (bHLH and WRKY).

### miRNA-Mediated Gene Regulation of Drought Tolerance

Plants respond to several environmental stresses, among which drought is the major stress that limits the yield of many crops. Regulation of gene expression through miRNA and its target complementarity has made plants that tolerate drastic effects caused by drought. In our study, different categories of targets were identified across millets as being specific to that particular family of miRNA. These include different enzymes, such as protein kinases, peroxidase, amino/carboxy peptidases, stress-associated proteins (HSPs, RNA binding proteins), and drought-specific TFs (ARF, NAC family, MAD box, WRKY, bHLH, and ZFs). The important miRNA families related to drought along with the millet-specific target genes are presented in Tables 3A, 3B.

**Table 3A:** Species-specific microRNA families associated with drought-responsive traits.

**miRNA**	**Stress-related mRNAs function**	**Species**
miR104, miR105, miR1120, miR123, miR150, miR154, miR158, miR163, miR38, miR41, miR6, miR156, miR171, miR2108, miR164, miR395, miR167, miR170, miR160, miR319, miR169, miR172, miR165	Dehydration stress	Foxtail millet
miR396	Dehydration stress	Sorghum
miR1873, miR10095	Drought stress	Finger millet
miR399	Drought stress	Foxtail millet
miR160, miR164, miR167, miR169, miR171	Drought stress	Pearl millet
miR165, miR395	Drought stress	Pearl millet, proso millet
miR2108, miR1432, miR159, miR160, miR162, miR168, miR169, miR319, miR390, miR393, miR408, miR167, miR156, miR171, miR172, miR399	Drought stress	Proso millet
miR167, miR10294, miR156, miR169, miR156, miR396	Drought stress	Sorghum
miR10290	Drought stress, oxidative stress, water-deficit stress	Finger millet
miR11947	Drought stress, oxidatively stressed leaves, water-deficit stress	Pearl millet
miR10110, miR10133, miR10157, miR10376	Drought stress, oxidatively stressed leaves, water-deficit stress	Sorghum
miR2108, miR91	Drought stress, water-deficit stress	Finger millet
miR156, miR169, miR160, miR10403	Oxidative stress	Sorghum
miR167, miR77	Oxidative stress, water-deficit stress	Sorghum
miR845, miR8770, miR7748	Water-deficit stress	Finger millet
miR156	Water-deficit stress	Sorghum

**Table 3B:** Species-specific microRNA families associated with drought-responsive genes, transcription factors, and other molecules.

**miRNA**	**Drought-responsive genes, transcription factors, and enzymes**	**Species**
miR156	ARF, E2F, HSP, nst1, EAT1, EMB1444, LRBPK, BIG, bHLH, WRKY, HIPP, SRPK, kinase byr2-like, SAUR, BRG3, LAZ1, ANK, GLYR1, NCA1, NRT1/PTR, Znf, STK	Foxtail millet
miR160	Serine carboxypeptidase, KN1, YUCCA4, STK, NSP1, Znf, WRKY, XET, ARF	
miR162	BIG, 14-3-3-protein, NAC, HSP, STK, SRPK, GrpE	
miR164	Carboxylesterase, F-box, GAUT, ANK, GLDC, HPR3, TLC, DMP, LRR receptor, NRT1/PTR, PBL, STK, bHLH, NAC, SRPK, Znf, SWI/SNF, syntaxin, BIG, LAMP1, WSD1, WRKY, XET, 11S globulin seed storage protein	
miR165	AMOT, ARF, NDR1/HIN1, GSTU6, PBL, Znf, WNK1, parC, STK, TIP41, LRR receptor, carboxylesterase, WRKY, NAC	
miR167	LRBPK, LAZ1, Znf, IAA, NRT1/PTR, ARF, NAC, HS1, LRR receptor, SRPK, SAC3, PBL, STK	
miR168	bHLH, carboxylesterase, ARF, NAC, serine carboxypeptidase, DMP, NRT1/PTR	
miR169	12-oxophytodienoate reductase, NAC, NDR1/HIN1, PIX, STK, serine carboxypeptidase, YLS3, YUCCA4, BAT1, GSTU6, HHL1, LRR receptor, PLS1, Znf, nst1, LAMP1, RsgI6	
miR170	GAUT, argininosuccinate lyase, NAC, RsgI6	
miR171	ANK, ARF, SAUR, carboxylesterase, LRBPK, SKIP35, SPIKE 1, Znf, LRR receptor, RIBA 2, GAUT, NRT1/ PTR	
miR172	ARF, bHLH, Znf, STK	
miR2108	GAUT, TPD1, STK, ARF, HSP, LRR receptor, NRT1/ PTR, ANK, Znf, SKIP35, TSJT1	
miR319	Carboxylesterase, kinase byr2-like, NDR1/HIN1, aminotransferase, SRPK, PMT, Znf, ITN1	
miR397	Znf, auxin, HSP, LRR receptor	
miR399	LRBPK, NAC, syntaxin, TBC1, MKS1, WRKY, DPH, Znf, PBL, SAUR, STK	
miR10347	HSP	Pearl millet
miR155	Cu-Zn superoxide dismutase, eIF-4A	
miR156	Glutathione S-transferase mRNA, HSP, RCI2A	
miR159	Purothionin, NBS-LRR, superoxide dismutase, VATPc, protein kinase	
miR44	ClpX1, NBS-LRR	
miR156	LOV	
miR164	NCED1	
miR167	Alanine aminotransferase, aspartate aminotransferase, LOV	
miR168	Dehydrin mRNA	
miR172	LOV, aspartate aminotransferase	
miR399	NCED1, LOV, aspartate aminotransferase, KN1	
miR10403	WRKY, Oxidatively stressed	Sorghum
miR156	Cyclin-T1-4, F-box, FERONIA, STK, FERONIA, GRX11, NKAP, HSP, cyclin-T1-4, F-box, MEL1, methylmalonate-semialdehyde dehydrogenase, PBL, PHOX4, RALF, RIC10, RING-H2 finger	
miR160	ARF, ACT domain-containing protein ACR4, aspartyl protease, HSP, HKT8, L-ascorbate oxidase, LECRK91, LRBP, CP12, F-box, NIR1, MYOB, NCS, NRT1/ PTR, TBL	
miR167	Agmatine deiminase, arogenate dehydrogenase 2, HSP	
miR167	ANK, ARF	
miR169	BoGH3B, flotillin-like protein 2, MAN2A, alpha-taxilin, ATG6, METTL13, MYOB, STK LECRK4, HSP, WRKY, aconitate hydratase, arginine/serine-rich protein	
miR396	ARF, NEP1, IAA, Lr10, NBR, KN1, LRR receptor, NRT1/ PTR, nst1, bHLH, disulfide isomerase, WRKY, YLS7, BRG3, XET, E2F	

During the stress conditions, excess concentration of ROS is accumulated within the cells, resulting in cellular oxidative damage. Studies have shown the involvement of peroxidase in ROS scavenging under drought-stressed conditions. In sorghum, the peroxidase family targeting six miRNA families under drought conditions were reported (Katiyar et al., 2015). We have also found sorghum (sbi-miR160), foxtail millet (sit-miR169p, sit-miR171n, and sit-miR395c), and pearl millet (pgl-miR156) miRNAs regulating different peroxidase families. Plants exposed to drought or heat stress produce HSPs, which play a crucial role in protecting from stress conditions (Wang et al., 2004). They function as molecular chaperones, facilitating in folding of proteins, which is important for plants to cope with drought stress (Ford et al., 2011). We found miRNAs pgl-miR156 and pgl-miR10347 mediating HSPs. In sorghum, miRNA families targeting HSP were under down-regulation during drought stress (Katiyar et al., 2015).

Our study identified that the pmi-miR164, pmi-miR399 were involved in targeting NCED protein. It encodes 9-cis-epoxycarotenoid dioxygenase, which is negatively correlated with ABA accumulation, whereas in some species, its expression increased along with ABA accumulation under water stress (Changan et al., 2018). The sbi-miR164a (sorghum) and sit-miR2118d (foxtail millet) regulating protein NRT1/PTR family in *Arabidopsis* was suggested as a tolerance mechanism for abiotic stress through reallocation of nitrate to plant roots (Corratge-Faillie and Lacombe, 2017). The pgl-miR155 regulating Cu/ZnSOD transcripts found in pearl millet had an antioxidant protector system under water stress conditions in *L. corniculatus* (Borsani et al., 2001).

The sit-miR156, sit-miR164, sit-miR166, sit-miR167, sit-miR171, and sit-miR393 in foxtail millet and sbi-miR169 and sbi-miR156 in sorghum were regulating the serine–threonine protein kinases (STPKs). It is observed that the phosphorylation state of several protein kinases changes when exposed to drought stress, implying their regulation in the drought-response signaling pathway. A change in concentration of free amino acid also has an impact on induction of drought stress in many plants. In *Brassica* leaves, the activities of alanine aminotransferase and aspartate amino transferase led to an overall decrease in protein synthesis under drought stress (Good and Zaplachinski, 1994). The pmi-miR399, pmi-miR2118, and pmi-miR167 from proso millet, sbi-miR169 from sorghum, pgl-miR167 and pgl-miR528 from pearl millet, and sit-miR393, sit-miR396d, sit-miR397a, sit-miR156a, sit-miR160a, sit-miR164a, sit-miR166, and sit-miR172 from foxtail millet were found potentially regulating different aminotransferases.

It was observed that during drought stress, miRNA target genes code for specific TFs, which mediate the regulation of drought tolerance (Nepolean et al., 2014; Tang and Chu, 2017; Mittal et al., 2018). TF-mediated gene regulation includes several physiological and signaling pathways, such as abscisic acid (ABA)-mediated response, auxin signaling, osmotic, and antioxidant production (Ding et al., 2013). We found that many miRNAs are involved in TF-mediated gene regulation in different millet species in response to drought stress. NAC factors played an important role in drought tolerance through ABA signaling pathways (Wang et al., 2016; Aravind et al., 2017). It was found that NAC factors in foxtail millet was controlled by miR164. In rice, the overexpression of stress-responsive *NAC*1 (*SNAC*1) in guard cells reduced the transpiration losses by increased stomatal closure (Singh et al., 2015). ARFs are the key elements mediated by auxins, which contribute to the drought stress tolerance. In cowpea, the upregulation of miR160a and miR160b targeted different ARFs, thereby resulting in drought tolerance. We also observed that sit-miR160a-3p especially targeted several ARF family members, including ARF10, ARF13, ARF18, and ARF22. The sbi-miR167 and sbi-miR156 targeting ARFs were found to play a major role in the process of plant growth and development. A study on sweet potato revealed that IbARF5 increased the contents of carotenoids and enhanced drought tolerance in transgenic *Arabidopsis* (Kang et al., 2018).

The role of bHLH57 in tolerance to drought, salt, and oxidative stresses was identified in finger millet (Babitha et al., 2015). The up-regulation of miRNA targeting bHLH when cowpea was exposed to drought stress (Barrera-Figueroa et al., 2011) was also observed. The expression of bHLH122 was recorded under drought and osmotic stress conditions in *Arabidopsis* (Liu et al., 2014). We also identified the regulation of bHLH by sit-miR156a, sit-miR164a, sit-miR168, sit-miR172m, and sit-miR396e (foxtail millet) and sbi-miR160f (sorghum). ZF proteins were associated with different developmental and other stress responses (Golldack et al., 2011). The miRNAs pgl-miR164 in pearl millet, sbi-miR169d in sorghum, and sit-miR167d, sit-miR2118d, sit-miR399j, sit-miR397a, and sit-miR395c in foxtail millet targeting ZFs were identified from our comparative study. Under drought conditions, the down-regulation of miRNA vun_cand030, which targeted ZF, was recorded in cowpea (Barrera-Figueroa et al., 2011).

Nuclear factor Y (*NFY*) is a major TF induced at the time of drought as we found in foxtail millet, in which the sit-miR169 family targeted NFYA 4, 5, 7, and 10. Li et al. (2008) reported that the NFYA was induced by ABA-dependent manner during drought stress. In *Arabidopsis*, miR169 targeting NFYA was down-regulated, but in cowpea, it induced the expression under drought stress (Barrera-Figueroa et al., 2011). In tomato, over-expressing miR169c exhibited better tolerance to drought due to reduced stomata opening (Zhang et al., 2011).

Kelch repeat-containing F-box proteins are known to be involved in response to both biotic and abiotic stresses (Sun et al., 2010). Studies conducted in sorghum revealed that the presence of several drought-responsive miRNAs targeting Kelch repeat-containing F-box protein (Katiyar et al., 2015). The expression of many F-box proteins was also noticed in cowpea under drought stress (Jia et al., 2012). sbi-miR156a, sbi-miR160f, sbi-miR169d, and sit-miR156a, sit-miR395b, and sit-miR164a were found targeting F-BOX proteins in our study. WRKY acts as positive regulators of ABA signaling in several stress responses. Its involvement in heat stress in sunflower was controlled negatively by miR396 (Giacomelli et al., 2012). We have also identified sbi-miR169d, sit-miR156, sit-miR160a, sit-miR164, sit-miR396, and sit-miR166 targeting the WRKY.

We identified pgl-miR155, pgl-miR156 specific to MADS box proteins. Several studies have started to identify various members of the MADS-box gene family as an important molecular component involved in different types of stress responses. Studies showed that the MADS-box genes act as critical negative regulators of growth, improving plant survival, while others function as positive regulators of stress tolerance, associated with regulating the maintenance of primary metabolism, ABA signaling, ROS homeostasis, and detoxification processes through antioxidant enzymatic activities (Causier et al., 2002; Jia et al., 2018; Castelán-Muñoz et al., 2019; Zhao et al., 2020).

## Conclusion

Novel miRNAs were identified by exploring the genomic resources of pearl millet, sorghum, foxtail millet, finger millet, and proso millet, and by comparing the miRNAs among millet species through a series of *in silico* approaches. Structural and functional classification of the identified miRNAs explained the unique and common features among the five millet species. The gene targets of miRNA were identified, and based on the GO annotation, they were classified into several functional groups. The drought-responsive gene targets regulated by the miRNAs were identified, and their role in drought tolerance were comprehensively explained. The genes can be further explored in trait improvement programs to enhance the productivity of millets in arid and drought-prone ecologies. Considering the conservative nature of miRNAs, the results of our experiment can also be used in other crops to understand the drought stress mechanism.

## Data Availability Statement

The datasets presented in this study can be found in online repositories. The names of the repository/repositories and accession number(s) can be found in the article/Supplementary Material.

## Author Contributions

NT planned and designed the research. AC, AV, RM, and AR conducted the experiments and data analysis. All authors contributed equally to the manuscript preparation, read, and approved the final manuscript.

## Conflict of Interest

The authors declare that the research was conducted in the absence of any commercial or financial relationships that could be construed as a potential conflict of interest.
